# Penetrating keratoplasty combined with intrascleral fixation of a four-haptic intraocular lens in aphakic eyes with corneal pathology

**DOI:** 10.3389/fmed.2026.1861508

**Published:** 2026-06-26

**Authors:** Chang Xu, Shuying Liao, Wei Wang, Zhuo Chen, Xiaochun Mao, Guigang Li

**Affiliations:** 1 Department of Ophthalmology, Tongji Hospital, Tongji Medical College, Huazhong University of Science and Technology, Wuhan, China; 2 Department of Ophthalmology, Xiangyang Central Hospital, Xiangyang, China; 3 Hubei Key Laboratory of Otolaryngologic and Ophthalmic Diseases, Tongji Hospital Affiliated to Tongji Medical College, Huazhong University of Science and Technology, Wuhan, China

**Keywords:** aphakia, clinical outcomes, corneal transplantation, four-haptic intraocular lens, penetrating keratoplasty, scleral fixation

## Abstract

**Purpose:**

To describe the surgical technique and evaluate the preliminary short-term outcomes of penetrating keratoplasty (PKP) combined with intrascleral fixation of a four-haptic intraocular lens (IOL) in aphakic eyes with corneal pathology and insufficient capsular support.

**Methods:**

This prospective case series included five consecutive patients who underwent combined PKP and intrascleral fixation of a four-haptic IOL. Four eyes had bullous keratopathy with aphakia, and one had traumatic corneal leukoma with aphakia. Best-corrected visual acuity (BCVA), intraocular pressure (IOP), graft clarity, corneal endothelial cell density (ECD), IOL position, and complications were assessed over at least 6 months.

**Results:**

Mean BCVA improved from 3.00 ± 0.00 logMAR preoperatively to 1.04 ± 0.14 logMAR at 6 months (*t* = 14.37, *p* < 0.001). Mean IOP changed from 15.66 ± 1.98 mmHg to 16.48 ± 0.64 mmHg, without significant difference (*t* = 0.473, *p* = 0.661). No major intraoperative complications occurred. Four of five grafts remained clear. In the four measurable cases, mean ECD at 6 months was 2,445 ± 193.5 cells/mm^2^. All IOLs remained centered and stable.

**Conclusion:**

Combined PKP and intrascleral fixation of a four-haptic IOL appears to be a feasible single-stage option for selected aphakic eyes with corneal pathology and insufficient capsular support. Favorable short-term anatomical and visual outcomes were observed, but the small sample size, lack of control group, and short follow-up limit generalizability. Larger comparative studies with longer follow-up are needed.

## 1 Introduction

Corneal blindness remains a major cause of visual impairment worldwide (1, 2). Although lamellar keratoplasty has increasingly replaced penetrating keratoplasty (PKP) in selected corneal disorders, PKP is still required in eyes with complex full-thickness corneal pathology (3). In some patients, corneal opacity coexists with aphakia and insufficient capsular support, making visual rehabilitation particularly challenging (4). In these complex eyes, surgical treatment must address both restoration of corneal transparency and stable intraocular lens (IOL) fixation.

For aphakic eyes without adequate capsular support, secondary intraocular lens (IOL) implantation options include anterior chamber IOLs, iris-fixated IOLs, and scleral-fixated posterior chamber IOLs (5). Among these approaches, scleral fixation of a posterior chamber IOL provides a more physiological lens position and may offer anatomical and optical advantages (6–8). However, eyes requiring simultaneous corneal transplantation and secondary IOL implantation often have additional challenges, such as previous trauma, anterior synechiae, iris defects, previous vitrectomy, or graft failure, which may limit the applicability of some fixation techniques.

Despite these available approaches, the optimal strategy for eyes requiring both corneal transplantation and secondary IOL implantation remains under debate (9, 10). Although sutured scleral-fixated IOLs and several sutureless fixation techniques have been reported, clinical data regarding combined PKP and intrascleral fixation of a four-haptic IOL remain limited. The four-haptic IOL used in this study was selected because its fixation holes allow direct suture passage and intraoperative adjustment, which may help achieve stable centration in eyes without capsular support. In this prospective case series, we evaluated the preliminary short-term outcomes of combined PKP and intrascleral fixation of a four-haptic IOL in five consecutive aphakic eyes with severe corneal pathology and insufficient capsular support.

## 2 Methods

### 2.1 Study design and patients

This prospective case series included five consecutive patients who underwent combined PKP and intrascleral fixation of a four-haptic IOL at the Department of Ophthalmology, Tongji Hospital, Wuhan, China, between April 2020 and April 2021. The patients ranged in age from 29 to 56 years (mean, 45 ± 9.36 years); two were male and three were female. Four eyes were right eyes and one was a left eye. All eyes were aphakic and had either full-thickness central corneal opacity or corneal endothelial decompensation. Baseline clinical characteristics are summarized in Table 1.

**Table 1:** Clinical characteristics of the patients.

Case	Age (yr)/sex	R/L	Diagnosis	Procedure
1	50/F	R	Corneal transplant rejection, anterior iris adhesions, vitreous opacities, aphakia	PKP + IOL fixation, anterior synechiae release
2	41/M	R	Corneal endothelial decompensation, corneal leukoma, aphakia, post vitrectomy, post ocular trauma	PKP + IOL fixation
3	29/F	R	Corneal endothelial decompensation, aphakia, primary angle-closure glaucoma, microphthalmia	PKP + IOL fixation
4	49/M	R	Bullous keratitis, aphakic, iris defect	PKP + IOL fixation + pupiloplasty
5	56/F	L	Corneal leukoma, aphakia, eye rupture after debridement and suture, fixed dilated pupil	PKP + IOL fixation + pupiloplasty

F, female; M, male; R, right eye; L, left eye; PKP, penetrating keratoplasty.

### 2.2 Ophthalmic examinations

BCVA was measured using a standard visual acuity chart and converted to logMAR units for statistical analysis. IOP was measured using a non-contact tonometer (NIDEK NT-510, Japan) or an Icare tonometer. ECD was assessed by specular microscopy (NIDEK CEM-530, Japan). IOL power was determined using the IOL Master (Carl Zeiss Meditec AG, Germany), and axial length was measured by A/B-scan ultrasonography. Anterior segment optical coherence tomography (AS-OCT) was performed using the Visante OCT 1000 (Carl Zeiss, Germany).

### 2.3 IOL power calculation

A four-haptic aspheric IOL (Akreos Adapt AO, Bausch & Lomb, United States) was used in all cases and fixated to the sclera with 10–0 polypropylene sutures. IOL power was calculated using the SRK/T formula (A constant 118.40) based on the corneal curvature of the contralateral healthy eye and the axial length of the operative eye. The target postoperative refraction was approximately −0.50 D.

### 2.4 Surgical procedure

**Figure 1:**
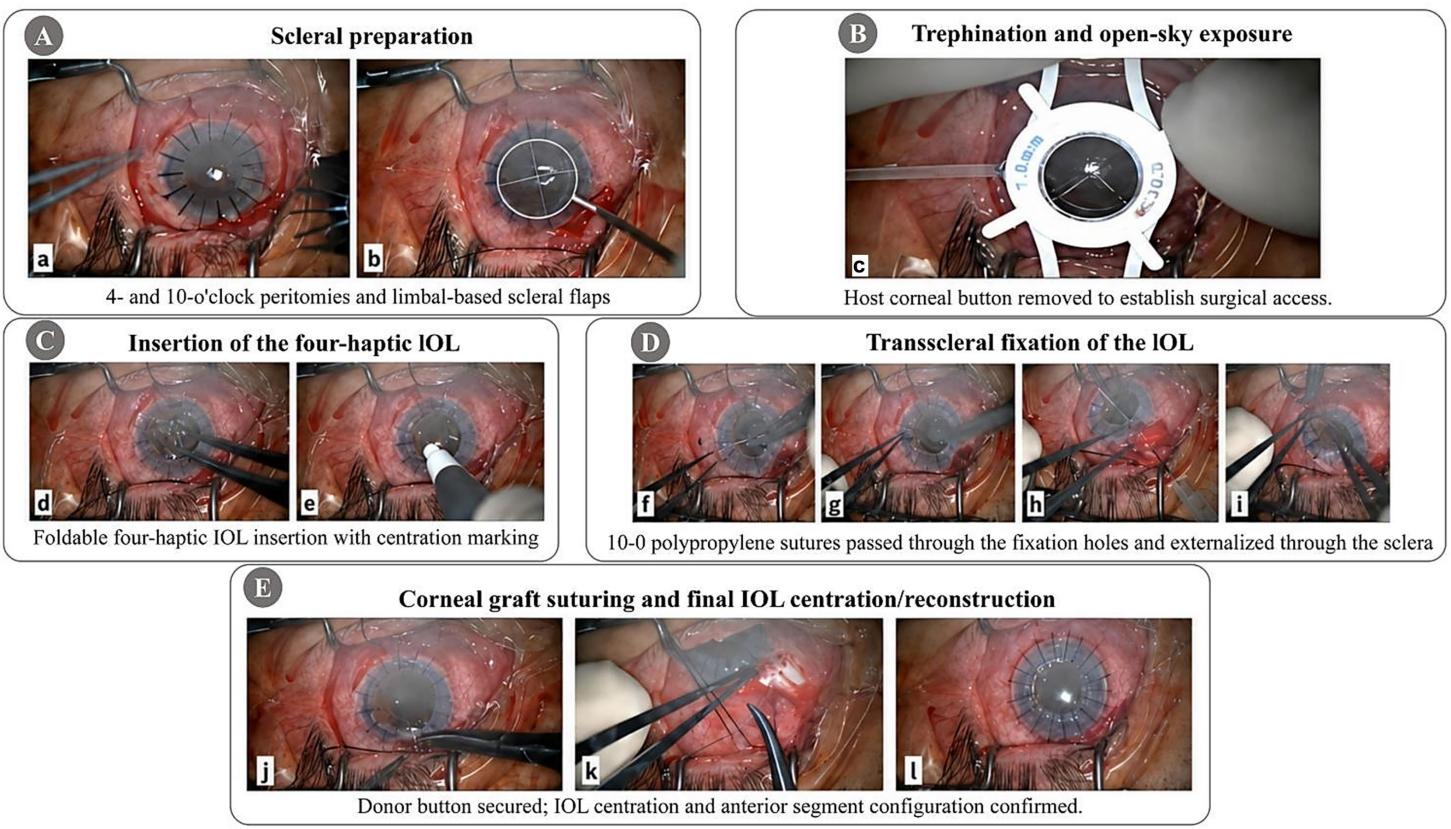
Schematic workflow of combined PKP and intrascleral fixation of a four-haptic IOL. **(A)** Scleral preparation: Corneal marks were denoted at 4- and 10 o’clock **(a)**. The corneal center was marked with a blue marker pen **(b)**. **(B)** Corneal trephination and open-sky exposure: Corneal negative pressure trephine suction fixation and drilling of the cornea **(c)**. **(C)** Insertion of the four-haptic IOL: Four-haptic aspheric intraocular lens **(d)**. Mark of the IOL center with blue marker pen **(e)**. **(D)** Transscleral fixation of the IOL: The 10-0 polypropylene suture thread is inserted into the sclera 2 mm behind the limbus, then passed through the top hole of four-haptic aspheric intraocular lens **(f)**. The 10-0 polypropylene suture was passed through the above hole **(g)**. Sutures made through the two holes were led out of the episclera using a syringe with a 30G needle through the sclera 2 mm from the limbus **(h)**. The same method was used to fix the opposite haptic of the intraocular lens **(i)**. **(E)** Corneal graft suturing and final reconstruction: The corneal graft was sutured intermittently **(j)**. The fixation of the intraocular lens is first passed through the superficial sclera and then knotted in alignment **(k)**. Final result of the surgery **(l)**.

All procedures were performed under general anesthesia. Briefly, conjunctival peritomies were created at the 4- and 10-o’clock positions, and two limbal-based partial-thickness scleral flaps measuring 3 × 3 mm were fashioned. Donor corneas were trephined at 8.0 mm, and recipient corneas were trephined 0.25 mm smaller. After removal of the diseased cornea, the IOL was inserted through the open-sky surgical field and secured to the sclera with 10–0 polypropylene sutures. The IOL center was marked intraoperatively to facilitate centration. Pupilloplasty was performed in two eyes when necessary to restore pupillary configuration and anterior segment anatomy (Figure 1).

### 2.5 Postoperative treatment and follow-up

Postoperatively, patients received topical corticosteroids, antibiotic eye drops, tacrolimus eye drops, and ocular surface supportive therapy according to a tapering regimen. Follow-up examinations were performed at 1, 3, and 6 months after surgery. BCVA, IOP, graft status, ECD, IOL position, and postoperative complications were recorded.

### 2.6 Statistical analysis

Data were analyzed using SPSS version 21.0. Quantitative variables are presented as mean ± standard deviation. Preoperative and postoperative BCVA and IOP were compared using paired *t*-tests. A *p*-value <0.05 was considered statistically significant.

## 3 Results

### 3.1 BCVA

**Figure 2:**
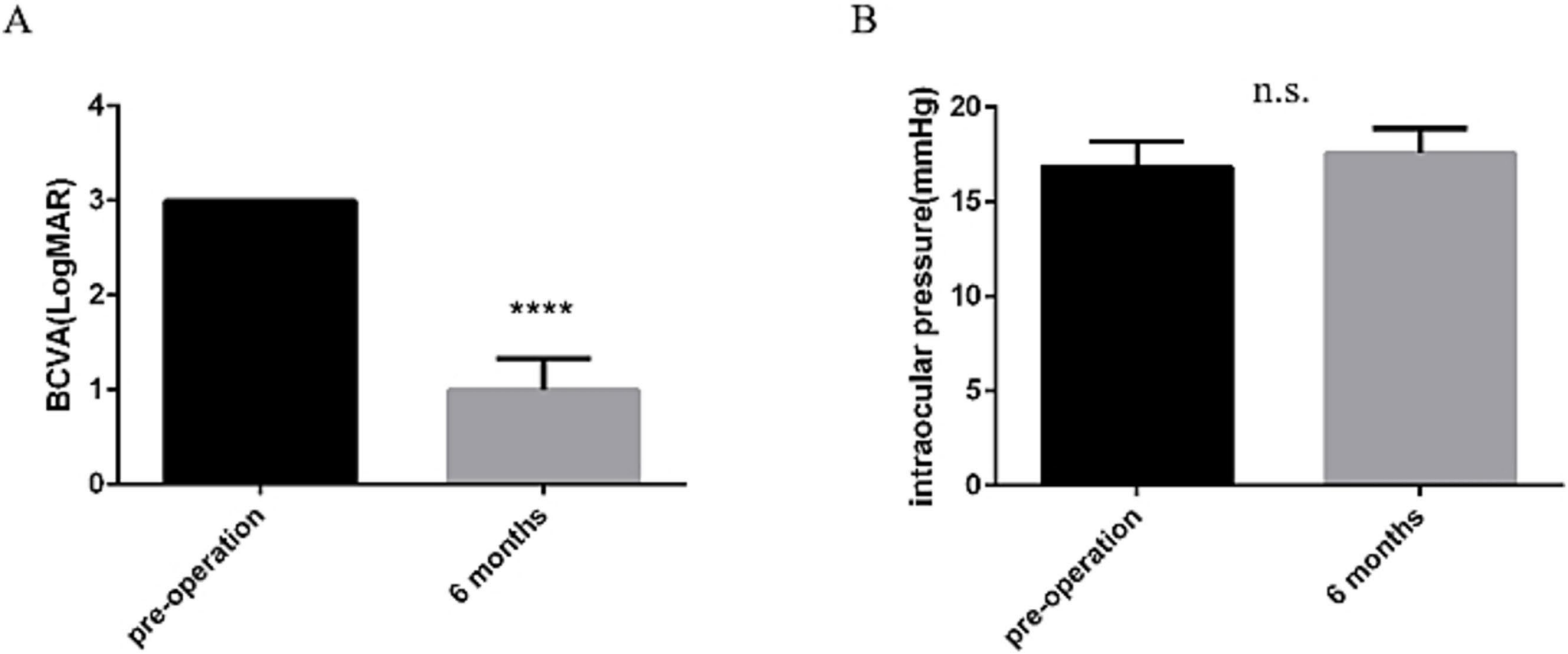
Changes in BCVA and IOP before and after surgery. **(A)** The best-corrected visual acuity (BCVA) was converted to LogMAR visual acuity. Comparison of visual acuity before and after surgery. **(B)** Comparison of intraocular pressure before and after surgery. Data are expressed as mean ± SD. ****p < 0.0001; ***p < 0.001; **p < 0.01; *p < 0.05.

As shown in Supplementary Table S1, both uncorrected visual acuity and best-corrected visual acuity (BCVA) improved postoperatively in all five patients. Before surgery, BCVA was 3.00 logMAR in all eyes, with a mean value of 3.00 ± 0.00 logMAR. At 6 months postoperatively, BCVA ranged from 0.50 to 3.00 logMAR, with a mean value of 1.04 ± 0.14 logMAR, representing a statistically significant improvement compared with baseline (*t* = 14.37, *p* < 0.001) (Figure 2A).

### 3.2 IOP

The mean preoperative IOP was 15.66 ± 1.98 mmHg, compared with 16.48 ± 0.64 mmHg at 6 months after surgery. This difference was not statistically significant (*t* = 0.473, *p* = 0.661; Figure 2B).

### 3.3 ECD

Four of the five grafts remained clear throughout follow-up. One graft became opaque because of immune rejection at 3 months postoperatively. ECD could not be measured in that eye at 6 months because of graft opacity. In the remaining four eyes, ECD ranged from 1927 to 3173 cells/mm^2^, with a mean value of 2445 ± 193.5 cells/mm^2^ (Supplementary Figure S1). No obvious decrease in ECD was observed between 3 and 6 months in the measurable cases.

### 3.4 AS-OCT and IOL position

AS-OCT demonstrated a smooth postoperative corneal surface and satisfactory anterior segment configuration (Supplementary Figure S2). All IOLs were well centered and remained stable during follow-up.

### 3.5 Complications

Intraoperative globe collapse occurred in two previously vitrectomized eyes with low ocular rigidity, but no intraoperative hemorrhage was observed. During follow-up, no retinal detachment or endophthalmitis occurred. Two patients developed loose sutures, which were removed without affecting graft-host healing. One patient developed graft opacity secondary to immune rejection despite systemic and topical corticosteroid treatment.

## 4 Discussion

The treatment of aphakic eyes with insufficient capsular support and corneal opacity remains a challenging task for most ophthalmologists today. Corneal laceration after ocular trauma, scarring after infectious keratitis, and long-standing bullous keratopathy in aphakic patients usually lead to full-thickness corneal transplantation (11). The most suitable method of secondary IOL implantation during PKP in an eye with insufficient capsular support is still debatable (12, 13).

Numerous options exist, including AC-IOLs, iris-sutured or iris-fixated IOLs, and sutured or sutureless scleral-fixated PC-IOLs. Being located closest to the natural lens position, PC-IOLs possess the inherent advantages of being close to the nodal point of the eye and also close to the eye’s rotational axis (13–15). However, each fixation technique has its own limitations, particularly in complex eyes with previous trauma, iris defects, prior vitrectomy, low ocular rigidity, or graft failure. Therefore, the selection of an IOL fixation method during PKP should be individualized according to ocular anatomy, surgical feasibility, and surgeon experience.

Several previous studies have reported outcomes of different IOL fixation techniques combined with PKP. Almulhim et al. (8) reported sutured scleral-fixated IOL implantation combined with PKP in 22 eyes and showed that this combined approach could improve visual function in a proportion of complex cases, although graft failure and the need for repeat transplantation remained important concerns during longer follow-up. This provides an important comparison for conventional sutured scleral fixation combined with PKP, especially in eyes with traumatic globe rupture, bullous keratopathy, failed graft, or corneal scarring.

Sutureless fixation techniques have also been explored. de Angelis et al. (16) reported PKP combined with sutureless scleral fixation using the Carlevale lens in vitrectomized eyes. They suggested that Carlevale lens implantation through transscleral plugs may reduce the duration of the open-globe stage, resulting in a potentially safer procedure, especially for vitrectomized eyes. This point is relevant to our study because globe collapse was observed in two previously vitrectomized eyes with low ocular rigidity.

Another technique, trocar-assisted sutureless scleral-fixated IOL implantation combined with PKP, has been reported by Karadag et al. (17). In their study, IOL stabilization was achieved without IOL-related complications during follow-up, suggesting that trocar-assisted sutureless fixation may be another useful option for eyes requiring PKP and secondary IOL fixation. Compared with these sutureless approaches, our technique uses a four-haptic IOL fixed with 10–0 polypropylene sutures through the haptic fixation holes. Although all IOLs remained centered and stable in the present series, the small sample size and short follow-up do not allow direct comparison of this method with other techniques.

We report the preliminary short-term outcomes of PKP and intrascleral fixation of a four-haptic IOL as a combined procedure to treat corneal disease and aphakia in vitrectomized eyes, eyes with bullous keratopathy, graft failure, and traumatic eyes.

In our study, all surgeries were performed by an experienced doctor under general anesthesia to reduce the risk of eye movement during surgery. Intravenous 20% mannitol (1-2 mg/kg) was administered 1 hour before surgery to reduce the risk of vitreous pressure elevation.

At the beginning of the operation, a 3 mm-long conjunctival peritomy was made at the 4 and 10 o’clock positions. A 3 × 3 mm partial-thickness limbal-based scleral flap was created after limited peritomy. We marked the center position of the IOL with a blue marker pen before implantation. During the “open-sky” period of PKP, a straight needle was passed ab externo and retrieved from the opposite scleral bed with a hollow needle. The suture was passed through two separate holes in the haptic before fixation. The four-haptic IOL is soft and can be easily adjusted to the optimal position, when necessary, by fastening or loosening the suture knot. At the same time, the decision to perform pupilloplasty is best undertaken intraoperatively after assessing the condition of the residual iris (18, 19).

Although our patients had undergone multiple previous ocular surgeries, including corneoscleral tear repair, complicated cataract surgery, intraocular foreign body removal, and had conditions such as primary angle-closure glaucoma with microphthalmia and graft failure, the outcomes showed that all five patients had improved visual acuity. Only one graft developed recurrent edema owing to immune rejection, and the ECD was not measurable. Globe collapse was observed in two patients, both of whom had eyes with low ocular rigidity after vitrectomy. However, no bleeding, retinal detachment, or infection was reported during the follow-up period. These findings suggest that this combined procedure may be feasible in selected complex eyes, but they should be interpreted cautiously because of the limited number of cases and short follow-up period.

PKP combined with intrascleral fixation of a four-haptic IOL may be considered as a single-stage surgical option for selected aphakic eyes with coexisting corneal pathology and insufficient capsular support. Penetrating keratoplasty (PKP) combined with intrascleral fixation of a four-haptic intraocular lens (IOL) is a well-established and effective surgical treatment (20, 21). In these cases, PKP is essential to restore corneal transparency through corneal transplantation while providing direct access for IOL implantation during the open-sky stage. This approach also reduces the need for additional incisions or surgeries, avoiding extra costs and potentially shortening patient rehabilitation. However, based on the present small case series, this technique should not be regarded as superior to other reported methods, including sutured scleral-fixated IOL implantation, Carlevale lens implantation, or trocar-assisted sutureless scleral fixation.

The pupil should remain constricted during PKP to protect intraocular tissues, especially against vitreous prolapse. An important advantage of the use of this lens in the open-sky setting is that the four-haptic IOL is soft and foldable, so the pupil does not need to be dilated. This may be useful in eyes with iris defects, anterior synechiae, or unstable anterior segment anatomy, although further comparative studies are needed to confirm its clinical advantages.

This study has several limitations. First, the sample size was very small, with only five eyes included, which limits statistical power and restricts the generalizability of the findings. Second, there was no control group, so direct comparison with other IOL fixation techniques combined with PKP was not possible. Third, the follow-up period was limited to 6 months, which is relatively short for evaluating long-term graft survival, endothelial cell loss, IOL stability, suture-related complications, glaucoma, and late immune rejection. Therefore, the present results should be considered preliminary and descriptive.

In conclusion, our method may represent a feasible single-stage approach for restoring visual function in patients with aphakia and coexisting corneal disease. However, because of the small sample size, absence of a control group, and short follow-up period, these findings should be interpreted cautiously. Larger comparative studies with longer follow-up are required to evaluate the long-term safety, stability, graft outcomes, and clinical value of this technique.

## Data Availability

The original contributions presented in the study are included in the article/Supplementary material, further inquiries can be directed to the corresponding authors.
