# Supplementary material for: Penetrating keratoplasty combined with intrascleral fixation of a four-haptic intraocular lens in aphakic eyes with corneal pathology
Source: Front Med (Lausanne). 2026 Jun 26;13:1861508. doi: 10.3389/fmed.2026.1861508 (PMC13349821; doi:10.3389/fmed.2026.1861508)
Supplement: Supplementary file 1 [file Supplementary_file_1.DOCX]

**Supplementary Material**

Supplementary material for this article can be found online, including Supplementary Table S1. The individual BCVA data before and after surgery, Supplementary Figure S1. Corneal endothelial cell density measurements before and after surgery, Supplementary Figure S2. Anterior segment optical coherence tomography images before and after surgery.

| Case | Preoperative | 1 months | 3 months | 6 months |
| --- | --- | --- | --- | --- |
| 1 | HM/10cm | 0.06 | 0.1 | HM/40cm |
| 2 | HM/20cm | 0.02 | 0.15 | 0.25 |
| 3 | FC/10cm | 0.02 | 0.03 | 0.04 |
| 4 | HM/30cm | 0.08 | 0.1 | 0.12 |
| 5 | HM/50cm | 0.06 | 0.06 | 0.08 |
| HM, hand motion; FC, finger counting; cm, centimeters. | | | | |

Supplementary Table S1. Individual Best-Corrected Visual Acuity Before and After Surgery.


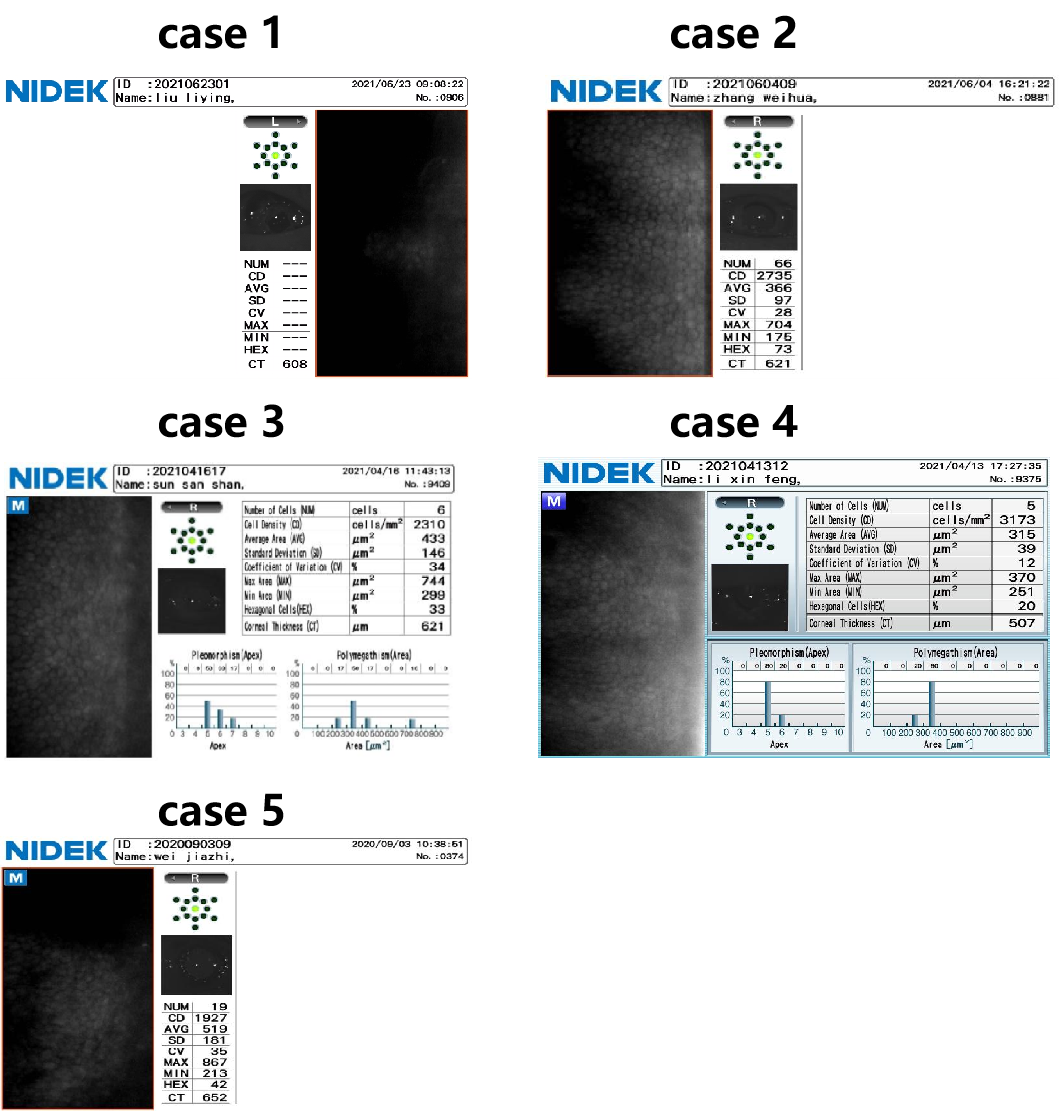


Supplementary Figure S1. Corneal endothelial cell density measurements before and after surgery. The corneal endothelium could not be measured in Case 5 because of graft opacity. The number of corneal endothelial cells was measured using NIDEK CEM-530 corneal endothelial cell counter (Japan).


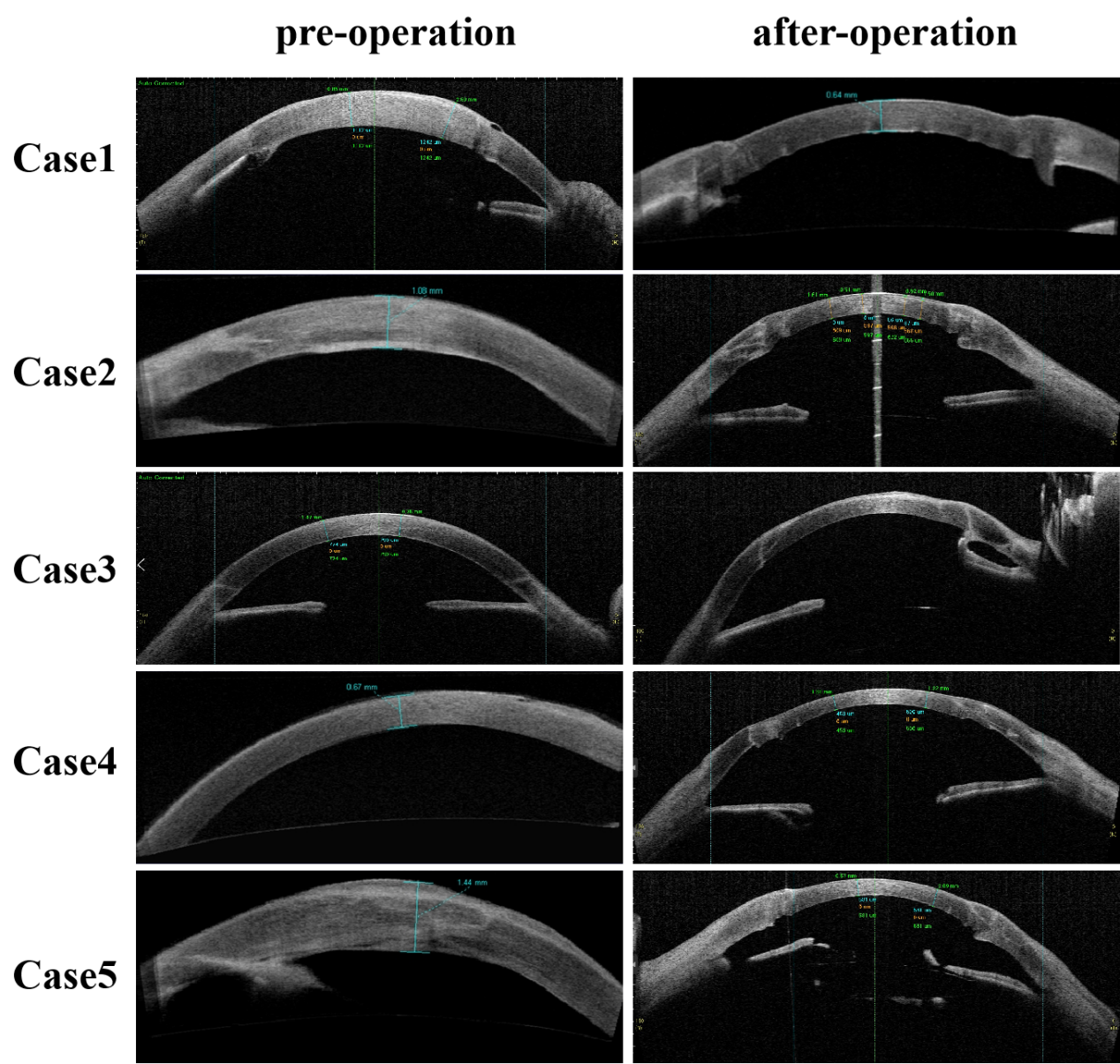


Supplementary Figure S2. Anterior segment optical coherence tomography images before and after surgery. The thickness of the central graft decreased after 6 months, and thus, the total central cornea was thinner than the preoperative cornea.
